# Gelam Honey Scavenges Peroxynitrite During the Immune Response

**DOI:** 10.3390/ijms130912113

**Published:** 2012-09-24

**Authors:** Mustafa Kassim, Marzida Mansor, Anwar Suhaimi, Gracie Ong, Kamaruddin Mohd Yusoff

**Affiliations:** 1 Department of Anesthesiology, Faculty of Medicine, University of Malaya, Kuala Lumpur 50603, Malaysia; E-Mails: marzida@gmail.com (M.M.); gracieo@um.edu.my (G.O.); 2 Department of Rehabilitation Medicine, Faculty of Medicine, University of Malaya, Kuala Lumpur 50603, Malaysia; E-Mail: anwars@um.edu.my; 3 Department of Molecular Biology and Genetics, Faculty of Arts and Science, Canik Basari University, Samsun 34083, Turkey; E-Mail: mykamar77@gmail.com

**Keywords:** inflammation, honey, nitric oxide, peroxynitrite, macrophage

## Abstract

Monocytes and macrophages are part of the first-line defense against bacterial, fungal, and viral infections during host immune responses; they express high levels of proinflammatory cytokines and cytotoxic molecules, including nitric oxide, reactive oxygen species, and their reaction product peroxynitrite. Peroxynitrite is a short-lived oxidant and a potent inducer of cell death. Honey, in addition to its well-known sweetening properties, is a natural antioxidant that has been used since ancient times in traditional medicine. We examined the ability of Gelam honey, derived from the Gelam tree (*Melaleuca* spp.), to scavenge peroxynitrite during immune responses mounted in the murine macrophage cell line RAW 264.7 when stimulated with lipopolysaccharide/interferon-γ (LPS/IFN-γ) and in LPS-treated rats. Gelam honey significantly improved the viability of LPS/IFN-γ-treated RAW 264.7 cells and inhibited nitric oxide production—similar to the effects observed with an inhibitor of inducible nitric oxide synthase (1400W). Furthermore, honey, but not 1400W, inhibited peroxynitrite production from the synthetic substrate 3-morpholinosydnonimine (SIN-1) and prevented the peroxynitrite-mediated conversion of dihydrorhodamine 123 to its fluorescent oxidation product rhodamine 123. Honey inhibited peroxynitrite synthesis in LPS-treated rats. Thus, honey may attenuate inflammatory responses that lead to cell damage and death, suggesting its therapeutic uses for several inflammatory disorders.

## 1. Introduction

Monocytes and macrophages play critical roles in innate immunity. Specifically, these cells act as a part of the first-line defense against bacterial, fungal, and viral infections during host immune responses [1] by expressing high levels of proinflammatory cytokines such as tumor necrosis factor (TNF)-α, interferon (IFN)-γ, and interleukin (IL)-1β, and cytotoxic molecules such as nitric oxide (NO) and reactive oxygen species (ROS) [2,3]. The inflammatory response is accompanied by the upregulation of a lipopolysaccharide (LPS)-inducible isoform of NO synthase (iNOS) [4], the expression of which is correlated with the degree of inflammation [5] as well as the presence of NO. However, in addition to their defensive roles, proinflammatory responses can damage DNA and other cellular structures, activating necrosis, apoptosis, and potentially, tumorigenesis [6,7].

Of the inflammatory modulators produced in response to LPS, NO is implicated in both acute and chronic models of inflammation, including septic shock [8], where there is extensive damage to host tissues. The damage is, in part, due to the reaction of NO with superoxide radicals, resulting in the formation of peroxynitrite (ONOO^−^). This short-lived oxidant species profoundly influences the inflammatory response at multiple cellular levels and is a potent inducer of cell death [9]. The biological targets of peroxynitrite include membrane, cytosolic, and nuclear receptors [9]. During inflammation, peroxynitrite also reacts with inflammatory mediators such as interleukins, and with iNOS, either triggering or enhancing proinflammatory pathways mediated by nuclear factor (NF)-κB [10]. Furthermore, the reaction products of peroxynitrite are detected in several pathological conditions, including vascular diseases, ischemia-reperfusion injury, circulatory shock, inflammation, pain, and neurodegeneration. Conversely, studies in animal models of inflammation and reperfusion injury have shown a protective effect for compounds that either inhibit peroxynitrite formation or accelerate its decomposition [9,11–16].

Honey is a well-known natural sweetener; this property is conferred by its complex mixture of carbohydrates, which are the main constituents of honey and are produced by honeybees from nectar sucrose. Monosaccharides, including fructose and glucose, are the major carbohydrates in honey; disaccharides (such as maltose and sucrose), trisaccharides (such as maltotriose and panose), and oligosaccharides are also present in honey [17–22]. In addition, honey also contains minerals, vitamins, enzymes, flavonoids, and phenolic compounds, making it a natural antioxidant [23]. Indeed, honey has been used in traditional medicine since ancient times [24]. It has potential effects in high oxidative stress conditions, and its ability to modulate antioxidant enzymes as well as its antioxidative properties provide protective effects against oxidative stress [25,26]. Recent studies demonstrated a strong correlation between the content of phenolic compounds in honeys from various floral sources and their antioxidant capacity and beneficial effects in human health [27–31]. One of the potential benefits of phenolic compounds is that they stabilize cell membranes by reducing lipid peroxidation and scavenging free radicals, [32] and they simultaneously enhance membrane integrity against several chemical and physical stress conditions [33]. Various studies investigated the potential protective effects of phenolic compounds against oxidative damage to red blood cells [34,35]. Honey demonstrated strong reducing activity against free radicals, with significant suppression/prevention of cell damage, complete inhibition of cell membrane oxidation and intracellular ROS production, and recovery of intracellular glutathione in cultured endothelial cells. This protective effect is mainly attributed to phenolic acids and flavonoids [36]. The phenolic compounds in honey were found to scavenge free radicals and prevent the production of malondialdehyde, a biomarker of oxidative damage, in a concentration-dependent manner [36,37].

Gelam honey is derived from the nectar of the Gelam tree (*Melaleuca* spp.), which grows in the forests of Malaysia. [38,39]. Gelam honey is reported to have a high phenolic content, with many phenolic compounds isolated from it, such as gallic acid, chlorogenic acid, caffeic acid, *p*-coumaric acid, ferulic acid, ellagic acid, quercetin, hesperetin, luteolin, kaempferol, and chrysin [40]. A previous study showed that Gelam honey exhibited antibacterial activity and free radical scavenging activity because of its phenolic compounds; furthermore, Gelam honey inhibited inflammation *in vitro* and *in vivo* [38–42]. It is also a potent inducer of heme oxygenase-1 (HO-1) and significantly reduces DNA damage and plasma malondialdehyde levels [41,43]. In this study, we tested the ability of Gelam honey to scavenge peroxynitrites in LPS/IFN-γ-stimulated murine macrophages (RAW 264.7) *in vitro*, and in a rat model of inflammation *in vivo*. Therefore, in this study, we carefully examined the cytoprotective effects of Gelam honey under the cellular damage conditions induced by two doses of LPS. In parallel, the effect of NO on murine macrophage (RAW 264.7) cell viability was also examined.

## 2. Results

### 2.1. Effect of Gelam Honey on Untreated and LPS/IFN-γ-Stimulated Cells

**Figure 1:**
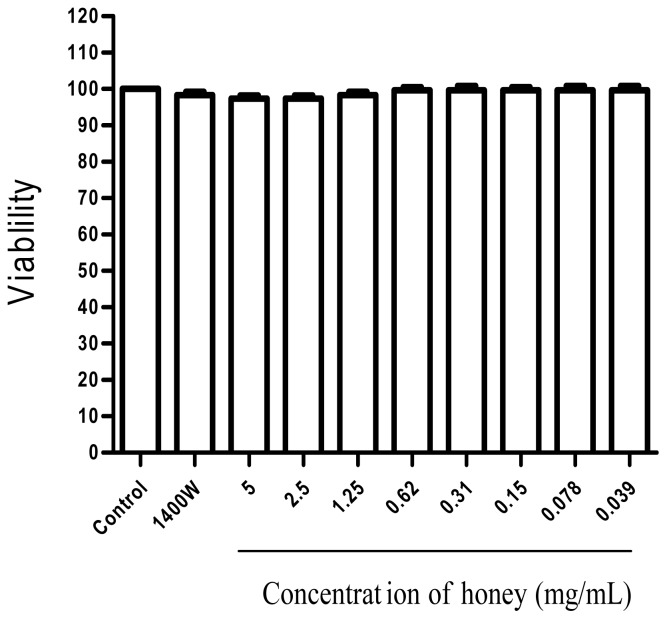
Effect of honey and the iNOS inhibitor 1400W on the viability of RAW 264.7 macrophages. Cells were treated or not treated (control) with the indicated concentrations of honey or 100 μM 1400W. Cell viability was determined by the mitochondrial reduction of 3-(4,5-dimethylthiazol-2-yl)-2,5-diphenyltetrazolium bromide (MTT). Data are expressed as the mean ± SEM of five independent experiments performed in triplicate. The viability of untreated cells was defined as 100%.

In RAW 264.7 cells, none of the doses of honey resulted in cytotoxicity, and the concentrations of honey did not affect cell viability (Figure 1). However, the viability of cells stimulated with 1 μg/mL LPS and 35 ng/mL IFN-γ was <68% of the control (untreated) value.

**Figure 2:**
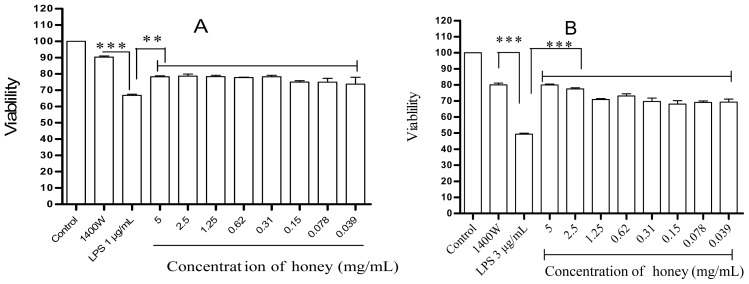
Cytoprotective effect of honey against LPS/IFN-γ-induced cytotoxicity. RAW 264.7 cells were incubated with either 1 μg/mL LPS and 35 ng/mL IFN-γ (**A**) or 3 μg/mL LPS and 35 ng/mL IFN-γ (**B**), and with various concentrations of honey or 100 μM of the iNOS inhibitor 1400W. The negative control was completely untreated (control), and the positive control was treated only with LPS/IFN-γ (LPS). After 24 h incubation, cell viability was determined using an MTT assay. *** *p* < 0.001 and ** *p* < 0.003 indicate significant differences compared with the LPS/IFN-γ group.

In contrast, as shown in Figure 2A, pretreatment with honey had a protective effect on LPS/IFN-γ-stimulated cells, significantly increasing their viability to >76% (*p* < 0.03), whereas the viability of cells pretreated with 1400W was >90% (*p* < 0.001). Increasing the LPS concentration to 3 μg/mL significantly reduced the viability of the untreated cells to <50% of the control value, whereas the addition of honey increased viability to >69% (*p* < 0.001), and the addition of 1400W resulted in >79% viability (*p* < 0.001) (Figure 2B).

**Figure 3:**
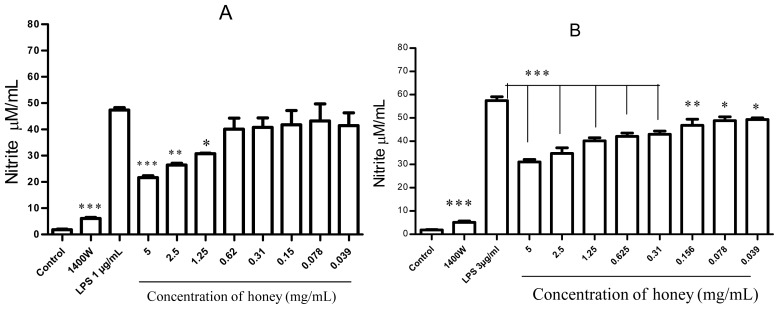
Effect of honey on NO production. NO production was estimated in RAW 264.7 macrophages pretreated for 1 h with the indicated concentrations of honey or the iNOS inhibitor 1400W (100 μM) and then exposed to 1 μg/mL LPS and 35 ng/mL IFN-γ (**A**) or 3 μg/mL LPS and 35 ng/mL IFN-γ (**B**) for 24 h. Nitrite accumulation in the supernatant was measured by the Griess reaction. All results are expressed as a percentage of the LPS/IFN-γ control (mean ± SEM of 5 independent experiments performed in duplicate). *** *p* < 0.001, ** p < 0.003, and * *p* < 0.05 indicate significant differences compared with the LPS/IFN-γ group.

Honey also significantly inhibited NO formation in cells that were stimulated with the indicated concentration of LPS (1 μg/mL or 3 μg/mL). A dose-dependent effect was observed at higher concentrations of honey (Figure 3).

### 2.2. Effect of Gelam Honey on Peroxynitrite in vitro and in vivo

**Figure 4:**
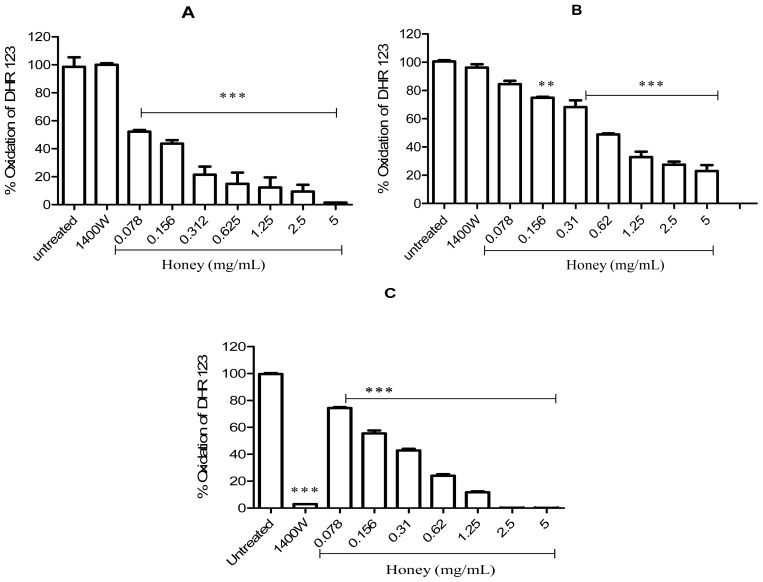
Effect of honey on the peroxynitrite-induced oxidation of DHR-123. (**A**) SIN-1, a peroxynitrite donor, was incubated for 2 h with different dilutions of honey (in PBS), 1400W (100 μM), and DHR-123, and the formation of rhodamine 123 was measured; (**B**) Honey (different dilutions in PBS) or 1400W (100 μM) were incubated for 15 min with DHR-123 and peroxynitrite, and the formation of rhodamine 123 was measured; (**C**) RAW 264.7 cells were incubated with honey (different dilutions in PBS), 1400W (100 μM), and DHR-123 for 60 min. Then, LPS/IFN-γ was added, and the cultures were incubated for an additional 24 h, after which the formation of rhodamine 123 was measured. Results are expressed as a percentage of the control (mean ± SEM of 3 independent experiments performed in triplicate). *** *p* < 0.001 and ** *p* < 0.003. Rhodamine 123 formation in the untreated control was defined as 100%.

An increase in the oxidation of dihydrorhodamine 123 (DHR-123) is indicative of the presence of peroxynitrite. In the 3-morpholinosydnonimine (SIN-1) model, honey was found to be a potent scavenger of peroxynitrite, inhibiting the SIN-1–induced oxidation of DHR-123 to rhodamine 123 with a half-maximal inhibitory concentration (IC_50_) of 0.148 mg/mL. Highly significant levels of oxidation were recorded in the presence of the iNOS inhibitor 1400W and in the (untreated) positive control (Figure 4A). To directly confirm the peroxynitrite scavenging activity of honey, peroxynitrite was incubated with or without different concentrations of honey (Figure 4B). In these experiments, honey also inhibited DHR-123 oxidation, presumably by scavenging peroxynitrite, with an IC_50_ of 0.68 mg/mL. However, oxidation was not inhibited by 1400W because the level of rhodamine 123 fluorescence was the same as that in the untreated control (Figure 4B). The addition of Gelam honey to RAW 264.7 cells that were induced with LPS/IFN-γ for 24 h also completely attenuated peroxynitrite activity and had an IC_50_ of 0.254 mg/mL (Figure 4C). In this experiment (unlike the previous 2 experiments), peroxynitrite synthesis was inhibited, and DHR-123 was not converted to its fluorescent product because cellular iNOS was blocked by 1400W (100 μM) (Figure 4C).

**Figure 5:**
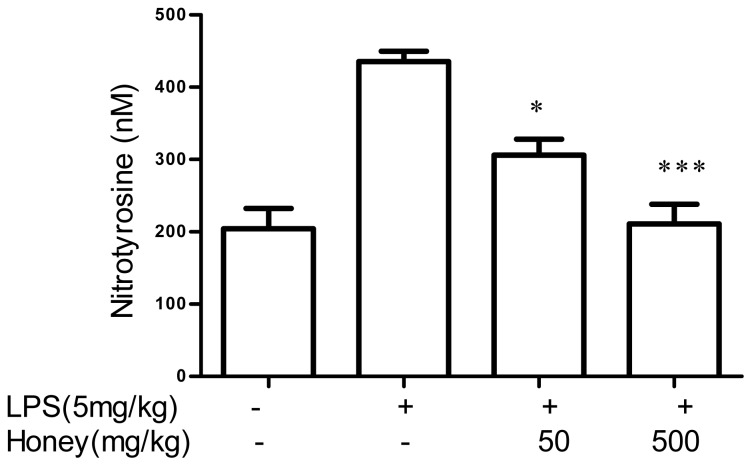
Effect of honey on the concentration of 3-nitrotyrosine in rat serum. Treated groups of animals were intravenously injected with either honey (50 or 500 mg/kg diluted in saline) or saline alone. One hour later, the treated animals were injected with LPS (5 mg/kg), and 4 h later, the treated and untreated rats were killed. The sera were collected and assayed for the presence of 3-nitrotyrosine. Data are expressed as the mean ± SEM. *** *p* < 0.001 and * *p* < 0.05 compared with the positive (LPS alone) control.

Pretreatment with 50 mg/kg or 500 mg/kg of honey also significantly inhibited peroxynitrite formation in rats, albeit not in a dose-dependent manner. Inhibition was determined by a reduction in 3-nitrotyrosine, which is an *in vitro* marker of peroxynitrite (Figure 5).

## 3. Discussion

This study investigated the ability of honey to modulate peroxynitrite-induced cell damage in LPS/IFN-γ-stimulated cultured macrophages and in a rat model of LPS-induced inflammation. The data presented here suggest that honey, through its antioxidant properties, is able to protect host immune cells from the inflammation-mediated cytotoxicity that develops in response to LPS stimulation. Gelam honey prevented the LPS-mediated decrease in RAW 264.7 cell viability caused by the high production of cytotoxic molecules such as cytokines, ROS, NO, and peroxynitrite [1]. Honey apparently scavenged peroxynitrite in RAW 264.7 cells induced with LPS/IFN-γ *in vitro*. Inhibitory effects were also observed upon incubation with the substrate SIN-1, a peroxynitrite donor, and with peroxynitrite itself. The ability of honey to scavenge peroxynitrite in tissues *in vivo* was demonstrated by the absence of 3-nitrotyrosine, a peroxynitrite marker, from rat serum.

Excess NO production is cytotoxic and has a broad spectrum of cellular effects. In RAW 264.7 macrophages, there was a pronounced increase in NO production and a significant decrease in cell viability after stimulation with LPS/IFN-γ, which is known to induce iNOS and thus increase cellular NO concentrations. This decrease in viability was much less pronounced in the presence of 1400W, suggesting that cytotoxicity was induced by iNOS and NO. In fact, NO production, measured as nitrite in the Griess reaction, was almost completely inhibited by iNOS inhibition.

The mechanism by which NO mediates toxicity includes the generation of reactive nitrogen derivatives such as peroxynitrite, which acts upon multiple cellular targets, including DNA and various proteins [44]. Thus, cellular exposure to high concentrations of peroxynitrite often leads to rapid, necrotic-type cell death due to acute and severe cellular energetic derangements [11,45,46]. In contrast, lower concentrations of peroxynitrite trigger delayed apoptosis that is mainly dependent on the activation of caspases 3, 2, 8, and 9, similar to other forms of oxidant/free radical-mediated apoptosis [47–50].

The effects of NO on cell viability are proportional to the cellular non-heme iron content. Thus, NO induces apoptosis in cells with a low non-heme iron level, such as RAW 264.7 macrophages, whereas it induces necrosis in cells with a high non-heme iron level, such as hepatocytes [51]. For example, NO induces both caspase-3 activation and cytochrome *c* release in apoptotic RAW 264.7 cells, and a caspase-3 inhibitor prevents NO-mediated RAW 264.7 apoptotic cell death [51]. NO readily reacts with non-heme iron to form iron-nitrosyl complexes [52,53], which are thought to protect cells from NO-induced toxicity by a scavenging mechanism [53]. Alternatively, NO may be converted to *S*-nitrosylating species, which act as potent regulatory molecules in a variety of cell types and cellular functions [54,55].

Recent studies show that peroxynitrite stimulates the release of the mitochondrial apoptosis-inducing factor, which subsequently triggers DNA fragmentation [56], release of mitochondrial pro-apoptotic factors, and cytochrome *c*-dependent apoptosis in the cytosol, through peroxynitrite-dependent oxidation of the mitochondrial permeability transition pore. The key role of peroxynitrite in promoting mitochondrial dysfunction is clearly exemplified in experimental sepsis, in which peroxynitrite production results in the inhibition of mitochondrial respiration in the diaphragm in a process associated with mitochondrial protein nitration. The latter is prevented by NO synthase inhibitors and Mn-porphyrin therapy [57]. Peroxynitrite-induced activation of the MLK/p38/JNK pathway also plays a crucial role in apoptosis [58–60]. This study shows that honey can inhibit NO production, and thus peroxynitrite formation, thereby reducing the effects of these cytotoxic compounds both *in vitro* and *in vivo*. Moreover, scavengers of peroxynitrite are known to be protective against tissue damage [9]. Some scavengers of peroxynitrite, such as uric acid, ebselen, mercaptoalkylguanidines, *N*-acetylcysteine, and dihydrolipoic acid, and some chemicals that work as decomposition catalysts of peroxynitrite, such as metalloporphyrins of iron and manganese, can attenuate the toxic effects of peroxynitrite *in vitro* and *in vivo* [11,16,61–68]. These compounds can reduce 3-nitrotyrosine immunoreactivity in various pathophysiological conditions and have beneficial effects in animal models of inflammation, sepsis, and reperfusion injury [57,68–77]. Many phenolic compounds, such as gallic acid, caffeic acid, kaempferol, ferulic acid, *p*-coumaric, and quercetin have been shown to inhibit peroxynitrite. Monohydroxylated phenolic compounds, such as ferulic acid and *p*-coumaric acid, act as peroxynitrite scavengers by nitration. On the other hand, compounds with a catechol moiety, such as caffeic acid and chlorogenic acid, reduce peroxynitrite by electron donation [66,78]. Interestingly, all of the above phenolic compounds were identified in Gelam honey [40]. Data from this study was in agreement with that of previous studies as described earlier. Moreover, a direct interaction through nitration and electron donation between honey and peroxynitrite is suggested by the results of the experiments involving SIN-1 and the direct incubation of honey and peroxynitrite. In addition, the presence of phenolic compounds in Gelam honey that act as potent scavengers of peroxynitrite, as described above, supports this hypothesis. In our previous studies, we demonstrated the anti-inflammatory activity of Gelam honey and its methanol and ethyl acetate extracts, on the basis of their abilities to suppress NO production in macrophages and rat inflammation models, inhibit the release of NO-induced cytokines (such as TNF-α, IL-1β, and IL-10) and high mobility group protein 1 (HMGB1), induces HO*-*1 in animal models, and protects organs from lethal doses of LPS that induce sepsis [39–41,79]. Gelam honey also inhibited iNOS protein expression in an animal model [80]. The results of this study support these previous findings, confirming the protective effects of honey mediated through the inhibition of NO and its derivatives (ONOO^−^) and, thus, its ability to prevent inflammatory-type cytotoxicity both in cultured macrophages and in animals.

The antioxidant and radical-scavenging abilities of Gelam honey are attributable to its phenolic compounds, which were also identified and quantified in previous studies [38]. The potential of Gelam honey to reduce LPS-induced inflammation is also mediated by its ability to reduce the release of proinflammatory cytokines and prostaglandin (PG) E_2_, both of which play a central role in inflammation [39,79]. In these studies, the reduced release of cytokines such as TNF-α, IL-1β, and IL-10 was quite dramatic in an endotoxemia model. This finding is particularly relevant to macrophages, where these mediators play a fundamental role in cell activation because they are released during the early stages of the inflammatory cascade [81]. In addition, honey may suppress NF-κB activation through other biologically active components, and consequently, inhibit iNOS induction [82,83]. Taken together, our work supports further investigations into the use of honey as a natural antioxidant, based on its ability to protect cells by inhibiting NO production, scavenging peroxynitrite, and modulating other inflammatory mediators such as PGE_2_, HMGB1, HO-1, and cytokines. The involvement of mechanisms other than iNOS inhibition, NO production, and peroxynitrite scavenging in these processes is suggested because low doses of honey had no effect on NO synthesis but did increase the viability of cells treated with LPS.

## 4. Experimental Section

### 4.1. Preparation of Gelam Honey

Fresh Malaysian honey, *Apis mellifera* (Gelam), was obtained from the National Apiary (Department of Agriculture, Parit Botak, Johor, Malaysia) and sent to the Malaysian Nuclear Agency for sterilization using a cobalt-60 source (model JS10000). Before its use in the *in vitro* and *in vivo* experiments described below, the sterilized honey was passed through a 0.20-μm filter syringe. The following concentrations were tested: 0.039, 0.078, 0.15, 0.31, 0.62, 1.25, 2.5, and 5 mg/mL.

### 4.2. Animals

Male Sprague–Dawley rats weighing 300–350 g were housed in individual cages under standard conditions (12 h light and 12 h dark). The animals were fed a diet of Purina lab chow and given water *ad libitum.* The study was carried out in accordance with the guidelines for animal experimentation of the University of Malaya Animal Ethics Committee and the protocols were approved under the terms set out: project license ANES/14/07/2010/MKAK (R).

### 4.3. Cell Culture and Reagents

The murine macrophage cell line RAW 264.7 was maintained in Dulbecco’s Modified Eagle Medium (DMEM), supplemented with 10% (*v*/*v*) heat-inactivated fetal bovine serum (FBS), 100 U/mL penicillin, and 100 U/mL streptomycin in a humidified 37 °C, 5% CO_2_ incubator. DMEM without phenol red, FBS, and antibiotics (penicillin, streptomycin) were purchased from Nacalai Tesque (Kyoto, Japan). LPS (*Escherichia coli* 0111 B4), iNOS inhibitor (1400W), and IFN-γ were purchased from Sigma-Aldrich (St. Louis, MO, USA).

### 4.4. LPS/IFN-γ Stimulation of RAW 264.7 Cells

RAW 264.7 cells were grown in 10% FBS-DMEM and seeded at a density of 2 × 10^6^ in a 24-well plate, followed by incubation for 24 h at 37 °C. The cells were then washed with PBS and resuspended in fresh medium containing different concentrations of Gelam honey (0.039–5 mg/mL) or 100 μM of 1400W. Untreated cells were included as positive or negative controls in each experiment. One hour later, 1 or 3 μg/mL of LPS and 35 ng/mL of IFN-γ were added to the cultures, followed by incubation for 24 h at 37 °C and 5% CO_2_ [84]. The cells were then processed for viability or NO or peroxynitrite detection as described below.

### 4.5. Measurement of Mitochondrial Respiration

The viability of RAW 264.7 macrophages was determined in cultures treated or not treated with LPS/IFN-γ and different concentrations of Gelam honey or 1400W. The viability was measured in terms of cellular respiration as assessed by the mitochondrial-dependent reduction of MTT to formazan. Cells were cultured, stimulated with LPS, and treated with honey or 1400W as described above, after which 100 μL of MTT (5 mg/mL) was added to each well, followed by 1 h incubation under the same conditions. The MTT solution was then removed and the cells were solubilized in 200 μL DMSO with shaking for 5 min. The absorbance was measured at 550 nm using a microplate reader (GloMax^®^-Multi Microplate detection; Promega, Madison, WI, USA) [85]. All experiments were repeated 5 times in triplicate.

### 4.6. Nitric Oxide Assay

Nitric oxide has a half-life of only a few seconds before it is quickly converted to nitrate and nitrite. These products can be measured using the colorimetric Griess reaction to indirectly determine the NO concentration. Therefore, RAW 264.7 cells were cultured and treated as described in Subsection 4.4 (LPS/IFN-γ stimulation of RAW 264.7 cells), after which 100 μL of the culture was placed in a 96-well plate, together with an equal amount of Griess reagent (50 μL of 1% sulfanilamide in 5% concentrated H_3_PO_4_ and 50 μL of 0.1% naphthylethylenediamine dihydrochloride in distilled water). The reaction between the Griess reagent and the nitrite present in the supernatant yields a pink derivative that can be spectrophotometrically quantified from a concentration curve prepared from a nitrite standard [40].

### 4.7. DHR-123 Oxidation Assay

#### 4.7.1. DHR-123 Oxidation Using SIN-1

SIN-1 spontaneously releases NO and superoxide under physiological conditions. At pH 7.4, SIN-1 is converted to SIN-1A via base-catalyzed ring opening. During ring opening, the oxygen undergoes univalent reduction to O_2_^−^. SIN-1A then releases NO and is converted to the stable metabolite SIN-1C, whereas the O_2_^−^ radical reacts with NO to form peroxynitrite (ONOO^−^). The oxidation of DHR-123 by ONOO^−^ results in the formation of fluorescent rhodamine 123, the amount of which can be measured by fluorometric analysis (GloMax^®^-Multi Microplate) at an excitation wavelength of 460–530 nm and an emission wavelength of 530–590 nm. In experiments examining the effects of honey on peroxynitrite scavenging, 100 μM of SIN-1 was used and the reactions were carried out in PBS, with the incubation of the samples for 2 h at 37 °C [84].

#### 4.7.2. DHR-123 Oxidation Using Peroxynitrite

The ability of peroxynitrite to oxidize DHR-123, thus converting it to rhodamine 123, was also measured directly as previously described [9]. Briefly, 10 μM peroxynitrite was mixed in PBS containing 20 μM DHR-123, in the absence or presence of either honey (0–5 mg/mL) or 100 μM of 1400W. After a 15-min incubation period at room temperature, the fluorescence of the rhodamine 123 reaction product was measured (GloMax^®^-Multi Microplate) at an excitation wavelength of 460–530 nm and an emission wavelength of 530–590 nm.

#### 4.7.3. DHR-123 Oxidation by LPS/IFN-γ-Treated RAW 264.7 Cells

Cells were cultured, stimulated with LPS, and treated with honey or 1400W as described in subsection 4.4 (LPS/IFN-γ stimulation of RAW 264.7 cells) in the presence of 10 μM DHR-123. After 24 h, 100 μL of the culture suspension was removed, and the amount of rhodamine 123 was determined fluorometrically.

### 4.8. Induction of an Immune Response in LPS-Stimulated Rats and the Effects of Honey

Rats were divided into four groups of six animals each. An immune response was induced in the animals in three of the four groups by intravenous injection of 5 mg/kg LPS (0111B4; Sigma) diluted in saline. One of these groups served as the positive control (LPS only), whereas the other two groups were intravenously injected with 50 or 500 mg/kg honey diluted in saline. The fourth (negative control) group was given saline only. All doses of LPS and honey were prepared immediately before injection, and 0.5 mL of the preparations were injected. The blood was collected (by cardiac puncture) from rats 4 h after the immune response was induced. The levels of 3-nitrotyrosine in the sera were measured using an ELISA kit according to the manufacturer’s protocol (Cell Biolabs Inc., San Diego, CA, USA).

### 4.9. Statistical Analysis

Student’s *t*-test and a non-parametric one-way ANOVA were used to determine the statistical significance of differences between the experimental and control groups, with *p* ≤ 0.05 considered to be statistically significant.

## 5. Conclusion

In conclusion, our data showed that honey is a potent peroxynitrite scavenger *in vitro* and *in vivo* that has cytoprotective effects against peroxynitrite-mediated cellular injury and death. Moreover, the preservation of cellular viability from peroxynitrite-mediated damage is critical to any consideration of the potential therapeutic value of peroxynitrite scavengers in many diseases, including inflammation and sepsis. This finding suggests that honey has therapeutic applications for a wide range of inflammatory disorders.
